# Anaphylatoxins orchestrate Th17 response via interactions between CD16^+^ monocytes and pleural mesothelial cells in tuberculous pleural effusion

**DOI:** 10.1371/journal.pntd.0009508

**Published:** 2021-07-08

**Authors:** Shuanglinzi Deng, Xinyue Hu, Lisha Luo, Wei Tang, Yuanyuan Jiang, Feifei Yin, Chengping Hu, Juntao Feng, Xiaozhao Li

**Affiliations:** 1 Department of Respiratory Medicine, Key Cite of National Clinical Research Center for Respiratory Disease, Xiangya Hospital, Central South University, Changsha, Hunan, China; 2 Department of nephrology, Xiangya Hospital, Central South University, Changsha, Hunan, China; Johns Hopkins University, UNITED STATES

## Abstract

The complement system is activated in tuberculous pleural effusion (TPE), with increased levels of the anaphylatoxins stimulating pleural mesothelial cells (PMCs) to secrete chemokines, which recruit nonclassical monocytes to the pleural cavity. The differentiation and recruitment of naive CD4^+^ T cells are induced by pleural cytokines and PMC-produced chemokines in TPE. However, it is unclear whether anaphylatoxins orchestrate CD4^+^ T cell response via interactions between PMCs and monocytes in TPE. In this study, CD16^+^ and CD16^-^ monocytes isolated from TPE patients were cocultured with PMCs pretreated with anaphylatoxins. After removing the PMCs, the conditioned monocytes were cocultured with CD4^+^ T cells. The levels of the cytokines were measured in PMCs and monocyte subsets treated separately with anaphylatoxins. The costimulatory molecules were assessed in conditioned monocyte subsets. Furthermore, CD4^+^ T cell response was evaluated in different coculture systems. The results indicated that anaphylatoxins induced PMCs and CD16^+^ monocytes to secrete abundant cytokines capable of only inducing Th17 expansion, but Th1 was feeble. In addition, costimulatory molecules were more highly expressed in CD16^+^ than in CD16^−^ monocytes isolated from TPE. The interactions between monocytes and PMCs enhanced the ability of PMCs and monocytes to produce cytokines and that of monocytes to express HLA-DR, CD40, CD80 and CD86, which synergistically induced Th17 expansion. In the above process, anaphylatoxins enhanced the interactions between monocytes and PMCs by increasing the level of the cytokines IL-1β, IL-6, IL-23 and upregulating the phenotype of CD40 and CD80 in CD16^+^ monocytes. Collectively, these data indicate that anaphylatoxins play a central role in orchestrating Th17 response mainly via interactions between CD16^+^ monocytes and PMCs in TPE.

## Author summary

Tuberculous pleural effusion is characterized by intense chronic accumulations of fluid and lymphocyte cells and monocytes/macrophages in the pleural space. Complement mediators play important roles in providing protection against Mycobacterium tuberculosis. Our results demonstrated that Mycobacterium tuberculosis infection induced the amplification of complement activation in TPE. Complement activation produces anaphylatoxins that induce PMCs and CD16^+^ monocytes to secrete abundant cytokines capable of only inducing Th17 expansion, but Th1 was feeble. In addition, costimulatory molecules were more highly expressed in CD16^+^ than in CD16^−^ monocytes isolated from TPE. The interactions between monocytes and PMCs enhanced the ability of PMCs and monocytes to produce cytokines and that of monocytes to express HLA-DR, CD40, CD80 and CD86, which synergistically induced Th17 expansion. In the above process, anaphylatoxins enhanced the interactions between monocytes and PMCs by increasing the level of the cytokines IL-1β, IL-6, IL-23 and upregulating the phenotype of CD40 and CD80 in CD16+ monocytes. In summary, these data highlighted the importance of anaphylatoxins and the innate immune system in eliciting pathogenic T cell responses in TPE and suggested that monocytes, especially the CD16^+^ subset, might be an efficient target for controlling inflammation.

## Introduction

Tuberculous pleural effusion (TPE), one of the common forms of extrapulmonary tuberculosis caused by *Mycobacterium tuberculosis* (*Mtb*) infection [1], is characterized by intense chronic accumulations of fluid and lymphocyte cells[2–4] and monocytes/macrophages [5], in the pleural space.

Complement mediators play important roles in providing protection against Mtb [6, 7]. Our previous study demonstrated that the complement system was activated in TPE [5]. There are three activation pathways resulting in the generation of multiple effector molecules, including the anaphylatoxins C3a and C5a [8, 9], which induce monocyte [10], T lymphocyte [11] and neutrophil [12] chemoattraction and activation through binding to the receptors C3aR and C5aR, respectively.

The complement system and monocytes have close interactions [13]. Our previous research found that C3a and C5a stimulated pleural mesothelial cells (PMCs) isolated from the pleural cavity to secrete the chemokines CCL2, CCL7, and CX3CL1, which recruited nonclassical monocytes (NCMs) [5].

PMCs, as active participants in the pleural injury process, are capable of secreting various cytokines, chemokines, and complement and antigen presentation-related proteins [5, 14–16]. During Mtb invasion, PMCs secrete not only T cell costimulators such as IL-1β, IL-6, and TNF-α[15, 16] but also chemokines to recruit inflammatory cells, such as monocytes [5] and CD4^+^ T cells [17, 18]. Ye and Ribera E et al. demonstrated that the percentages of CD4^+^ T cells, such as Th1, Th9, Th17, Th22 and Treg cells, in the pleural effusion were higher than those in the peripheral blood in patients with TPE, and some pleural cytokines including IL-1β, IL-4, IL-6, TNF-α, and TGF-β exhibited significantly elevated levels in TPE, which could drive the differentiation of naive CD4^+^ T cells into different Th cell lineages[17–19]. However, it is unclear where the above mentioned cytokines came from and whether complement activation can promote the secretion of these cytokines by PMCs and affect the response of CD4^+^ T cells. On the other hand, C3a significantly increases lipopolysaccharide (LPS)-mediated IL-1β production by human monocytes, which induces increased Th17 responses [20]. Similarly, our previous studies found that C3a and C5a stimulated IL-β, IL-17 and IL27 production in human CD14^+^CD16^+^ monocytes from TPE [5]. However, whether the cytokines secreted by monocytes affect the response of CD4^+^ T cells in TPE and whether C3a and C5a enhance the above effects are not clear.

Naive CD4^+^ T cells are induced to differentiate into different Th subsets by various signals, including surrounding cytokines and antigen-presenting cells (APCs). APCs required to express sufficient levels of costimulatory molecules, such as CD80 and CD86 and present antigens on HLA-DR molecules to trigger the activation of T cell receptors. Delphine et al. found that interactions between human bronchial epithelial cells and monocyte-derived dendritic cells can induce specific Th. They demonstrated through cell co-culture that damaged human bronchial epithelial cells with severe asthma induce the release of cytokines and chemokines, and produce incompletely mature monocyte-derived dendritic cells that only show upregulation of CD86 [21]. However, whether interactions between PMCs and monocytes regulates the response of CD4^+^ T cells has not been investigated in the context of TPE. Weaver et al. demonstrated that C5a receptor-deficient DCs treated with ovalbumin and a TLR2 ligand promoted the induction of Treg and Th17 cells because of low steady-state HLA-DR expression and an impaired ability to upregulate CD86 and CD40 expression in response to TLR2, which suggested that anaphylatoxins could regulate APC phenotypes and thus affect the differentiation of CD4^+^ T cells [22].

In view of the above considerations, this study aims to clarify whether C3a and C5a induce PMCs and CD16^+^ monocytes to express and secrete cytokines that can induce CD4^+^ T cell response, and enhance the effect of interactions between monocytes and PMCs on CD4^+^ T cell responsein TPE.

## Materials and methods

### Ethics statement

The study was approved by the Ethics Committee of Xiangya Hospital, Central South University(201703581), and verbal informed consent was obtained by all subjects. 30 patients with TPE and 20 heart failure patients with TE were enrolled at Xiangya Hospital, and pleural effusion or blood samples were obtained from these subjects (the clinical characteristics of TPE and TE subjects are summarized in S1 Table).

### Study subjects

All TPE patients were diagnosed, as evidenced by growth of Mtb from pleural fluid or by demonstration of granulomatous pleurisy on closed pleural biopsy specimen in the absence of any evidence of other granulomatous diseases. All TPE patients were anti-HIV antibody negative and after anti-tuberculosis chemotherapy, the resolution of TPE and clinical symptoms was observed.

The patients were excluded if they had undergone any invasive procedures directed into the pleural cavity or if any chest trauma within 3 months prior to their hospitalization, or if the existence of a pleural effusion of unknown origin. At the time of sample collection, none of the patients had received any anti tuberculosis therapy, corticosteroids, or other nonsteroidal anti-inflammatory drugs.

The heart failure diagnosis was made according to the European Society of Cardiology (ESC) guidelines by clinical examination, medical history review and echocardiographic examination by one of four experienced cardiologists. These patients were excluded if they had accepted any invasive procedures directed into the pleural cavity or any chest trauma within 3 months prior to their hospitalization, or the existence of a pleural effusion of unknown origin, or had any other systemic diseases, such as renal, hepatic or connective tissue diseases. None of the patients had a history of tuberculosis or pulmonary infection within the month prior to their hospitalization.

### Sample collection and process

Pleural tissue samples from TPE patients were obtained by thoracoscope biopsy and fixed in formaldehyde for immunohistochemistry analyses. 500 to 1000 ml pleural fluid samples were collected in heparin treated tubes for each subject using a standard thoracocentesis technique within 24 h after hospitalization. 10 ml peripheral blood was drawn simultaneously. Specimens were centrifuged at 1200 g for 10 min. The cell-free supernatants of the pleural fluid and serum were frozen at -80°C immediately after centrifuging for later determination of the anaphylatoxin concentrations. The cell pellets of the pleural fluid were resuspended in PBS, and mononuclear cells were isolated by Ficoll Paque-PLUS density gradient centrifugation (GE Healthcare, Little Chalfont, UK) to determine the monocyte and T cell subsets within 1 h.

### Immunohistochemistry

The paraffin-embedded 4 μm sections of formaldehyde-fixed pleural tissue were dewaxed by xylene, rehydrated in different gradient alcohol and washed by PBS. Then antigen retrieval was conducted in 10 mM sodium citrate solution (pH 6.0) for 20 minutes in a microwave. Endogenous peroxidase was blocked with 3% hydrogen peroxide in PBS for 20 min and nonspecific binding was blocked by incubation in diluted normal goat serum for 60 min. The antibodies were mouse anti-human C3a monoclonal antibody (ab37230, Abcam), mouse anti-human C5a monoclonal antibody (ab11877, Abcam), rabbit anti-human C3aR1(NBP2-15649, Novusbio) antibody and rabbit anti-human C5aR1 antibody (ab59390, Abcam) and diluted to the manufacturers’ recommended concentrations and applied for overnight incubation at 4°C. Antibody labeling was detected using an SP goat IgG kit (PV-6000, ZSGB-Bio, China) according to the manufacturer’s instructions. Chromogenic reactions were performed with DAB liquid (ZLI-9018, ZSGB-Bio, China), and counterstaining was performed with Mayer’s hematoxylin (ZSGB-Bio, China). The slides were viewed under an imaging fluorescent microscope (Olympus BX51; Olympus, Tokyo, Japan). As negative controls, primary antibodies were replaced with normal rabbit IgG or normal mouse IgG antibodies.

### Immunofluorescence staining

PMCs isolated from TPE and TE were seeded into slides at a density of 1 × 10^4^ cells/cm^2^. Adherent PMCs were fixed and permeabilized with 0.3% Triton X-100 in PBS for 15 min at room temperature and washing with PBS, slides were incubated with 10% goat serum in PBS at 4°C for 60 min. Then incubated at 4°C overnight with the appropriate primary antibody, which were mouse anti human antibodies recognizing C3a (ab37230, Abcam, concentration 1:100), C5a (ab11877, Abcam, concentration 1:200), and rabbit anti-human antibodies recognizing Calretinin (ab92341, Abcam, concentration 1:200), C3aR (NBP2-15649, Novusbio, concentration 1:200), C5aR1 (ab59390, Abcam, concentration 1:200), at the concentration recommended by the manufacturer. After washing, slides were incubated for 40 min at room temperature in the dark with the appropriate secondary antibody, which were an Alexa Fluor 594-labeled, affinity-purified goat anti mouse IgG antibody (ab150116, Abcam, concentration 1:500) and an Alexa Fluor 488-labeled, affinity-purified goat anti rabbit IgG antibody (ab150077, Abcam, concentration 1:500). DAPI mounting medium (Vector Laboratories, Burlingame, CA) was used for cell nuclei staining at 25°C for 10 min. Finally, slides were viewed under an imaging fluorescent microscope (Olympus BX51; Olympus, Tokyo, Japan).

### Flow cytometry

The expression markers in PMCs and monocytes from the TPE and TE were determined by flow cytometry using surface staining with anti-human-specific antibodies conjugated with Alexa Fluor647, APC, BV510, FITC, BV421, PE, PE-Cy7, APC. These human antibodies included anti C3aR (*hC3aRZ8)*,–CD88 (*S5/1)*,–CD14 (*M5E2)*,–CD16 (*3G8)*,–HLA-DR (*Tu39)*,–CD40 (*5C3)*,–CD80 (*L307*.*4)* and–CD86 (*2331)* antibodies, which were purchased from BD Biosciences or Biolegend.

For Th subsets cell detection, cells were suspended in RPMI 1640 (Gibco) and stimulated with phorbol myristate acetate (PMA, 50 ng/ml; Sigma) and ionomycin (1 mg/ml; Sigma) in an incubator (37°C, 5% CO^2^) for 5 h. Incubated with antibodies against the surface markers CD3 (HIT3a, BV510, BD Biosciences) and CD4 (RPA-T4, BB515, BD Biosciences) for 30 min in the dark at 4°C, and then permeabilized with Cytofix/Cytoperm (eBioscience) at 4°C for 30 min. Intracellular cytokines were stained with anti-human IFN-γ(B27, PerCP-Cy5.5, BD Biosciences) and anti-human IL-17A antibodies (N49-653, BV421, BD Biosciences). Flow cytometry data was collected with a BD FACS Canto II flow cytometer and analyzed using FlowJo 10.0 software.

### Cell isolation

The peripheral blood and pleural effusion were obtained from subjects. The peripheral blood mononuclear cells (PBMCs) and pleural effusion mononuclear cells (PFMCs), respectively, were isolated using a Ficoll gradient centrifugation protocol.

Mononuclear cells were identified within the singlet gate (FSC-A/FSC-H) by flow cytometry (BD Biosciences) and monocyte subsets were purified based on CD14 and CD16 staining.

For isolating PMCs, the cell pellets of the TPE were resuspended in DMEM (Gibco, Invitrogen, Carlsbad, CA) containing 20% heat inactivated fetal bovine serum (FBS, Gibco), 20 ng/ml epidermal growth factor (R&D Systems), and 50 mg/ml gentamycin. The cells were seeded into 25 cm^2^ flasks at a density of 1×10^4^ cells/cm^2^ and placed in an incubator at 37°C, 5% CO_2_. After 24 h, the monolayers were washed with PBS to remove nonadherent cells, and fresh media was added. The monolayers were monitored until confluent (7–10 d) and then trypsinized and subcultured for 5 to 6 passages. After each passage, the cells grew to confluence within 4–5 d. In general, PMCs could be maintained for 6 to 7 passages before they became senescent. PMCs were identified with an anti-calretinin monoclonal antibody (S1 Fig).

Naïve CD4^+^ T cells were isolated by MACS based on negative selection using the Naïve CD4^+^ T cell isolation kit II (Miltenyi Biotec, Order no. 130-094-131, Bergisch Gladbach, Germany) and monocytes were isolated by MACS based on positive selection using the CD14 (Miltenyi Biotec, Order no. 130-050-201, Bergisch Gladbach, Germany) and CD16 isolation kit II (Miltenyi Biotec, Order no. 130-045-701, Bergisch Gladbach, Germany) according to the manufacturer’s instructions. The purity of naïve CD4^+^CD45RA^+^ T cells, CD16^+^ monocytes and CD16^-^ monocytes were > 92%, as measured by flow cytometry (S1 Fig). Trypan blue staining and cell count are used to identify dead and living cells, and the percentage of live cells after MACS reached more than 90%.

### RNA Preparation and real-time quantitative PCR (RT-qPCR)

Total RNA was isolated using Trizol (Life Technologies, Ober-Olm, Germany) according to the manufacturer’s guidelines and then quantification of the RNA concentration with Nanodrop (Thermo Scientific, Darmstadt, Germany). RNA was reverse transcribed into cDNA using a Takara First Strand cDNA Synthesis kit (Ambion, Foster City, CA) and then were subjected to real-time qPCR analysis using Power SYBR Green (Applied Biosystems, ABI 7100, Darmstadt, Germany). Primer sequences are listed in S2 Table.

### Cytokine measurements

The concentrations of complement components and cytokines including C3a, C5a, IL-1β, IL-6, IL-12, IL-23, TNF-α and TGF-β, in both the pleural fluid, PMCs and monocytes culture supernatant were measured by enzyme-linked immunosorbent assay (ELISA) kits following the manufacturer’s protocols (All kits were purchased from Neobioscience Technology Co, Ltd, eBioscience or Elabscience).

### Effects of Mpt64, C3a, C5a, C3aRA and C5aRA on PMCs in vitro

PMCs isolated from TPE were incubated in serum-free medium for 24 h before treatment, and then in the presence of medium alone, or with Mpt64 (20 μg/ml, Goodhere Biotechnology, Hangzhou, China) for different times (0 h, 12 h, 24 h or 48 h) or with different doses of Mpt64 (0 μg/ml, 5 μg/ml, 10 μg/ml, or 20 μg/ml) for 24 h. In experiments, C3a (20 nM, A118, CompTech), C5a (10 nM, pro-2300-b, ProSpec-Tany), C3aRA (50 nM, SB290157, Calbiochem), C5aRA (20 nM, PMX53, 5473, R&D systems), C3aR nonpeptide agonist (C4494, 50 μM, SigmaAldrich) were added for 24h to evaluate the effect of anaphylatoxins on PMCs and monocytes. Anaphylatoxins receptors were measured by flow cytometry, total RNA was isolated for PCR and culture supernatant was harvested for ELISA. Before the experiment, MPT64, C3a, C5a C3aRA and C5aRA were treated by Pierce High Capacity Endotoxin Removal Spin Columns(Thermo Fisher Scientific, 88273) to ensure the removal of endotoxin in practice.

### Expansion of Th cells

Purified naïve CD4^+^ T cells (5 × 10^5^) were cultured in 1 ml of complete medium containing human IL-2 (2 ng/ml, 202-IL/CF, R&D Systems) in 48-well plates and stimulated with plate-bound anti-CD3 (2 μg/ml, 555329, BD Pharmingen) and soluble anti-CD28 monoclonal antibodies (2 μg/ml, 555726, BD Pharmingen) for 5 d. The exogenous cytokines used were IL-1β (20 ng/ml), IL-6 (100 ng/ml), IL-23 (100 ng/ml), and TNF-α (50 ng/ml). Recombinant human IL-1β, IL-6, and TNF-α were purchased from Peprotech.

### Cell coculture

The purified monocytes were incubated in RPMI 1640 supplemented with 10% heat-inactivated fetal calf serum, 100 U/mL penicillin, and 100 μg/mL streptomycin. To evaluate the effects of PMC-monocyte, each monocyte subset were cocultured with PMCs (ratio 3:1) for 24 h. To evaluate the effects of conditioned monocyte subsets on T-cell activation, each monocyte subset was cocultured with autologous naive CD4^+^ T cells (ratio 1:5) in the presence of anti-CD3 and anti-CD 28 antibodies for 5 days. The co-culture method used is direct culture, in which the two types of cells are incubated together in a six-well plate (NEST Biotechnology) for 24 hours or 5 days to make direct contact between the cells. Trypan blue staining and cell count are used to identify dead and living cells. The T cells viability reached more than 80% after 5 days co-culture, and the cell viability in the monocytes subsets and PMCs all reached more than 90% after 24h coculture.

### Statistics

Data is expressed as the mean ± SD. Student’s t test was used for comparisons of two groups. Comparisons of the data between different groups were performed using one-way ANOVA or the Kruskal-Wallis test. All statistical analyses were performed using GraphPad Prism 7.0 software (GraphPad Software Inc., La Jolla, CA). Experiments were repeated at least three times to ensure reproducibility, and differences were considered statistically significant at P values < 0.05.

## Results

### Anaphylatoxins and their receptors exhibited significantly increased in the PMCs of TPE patients

**Fig 1:**
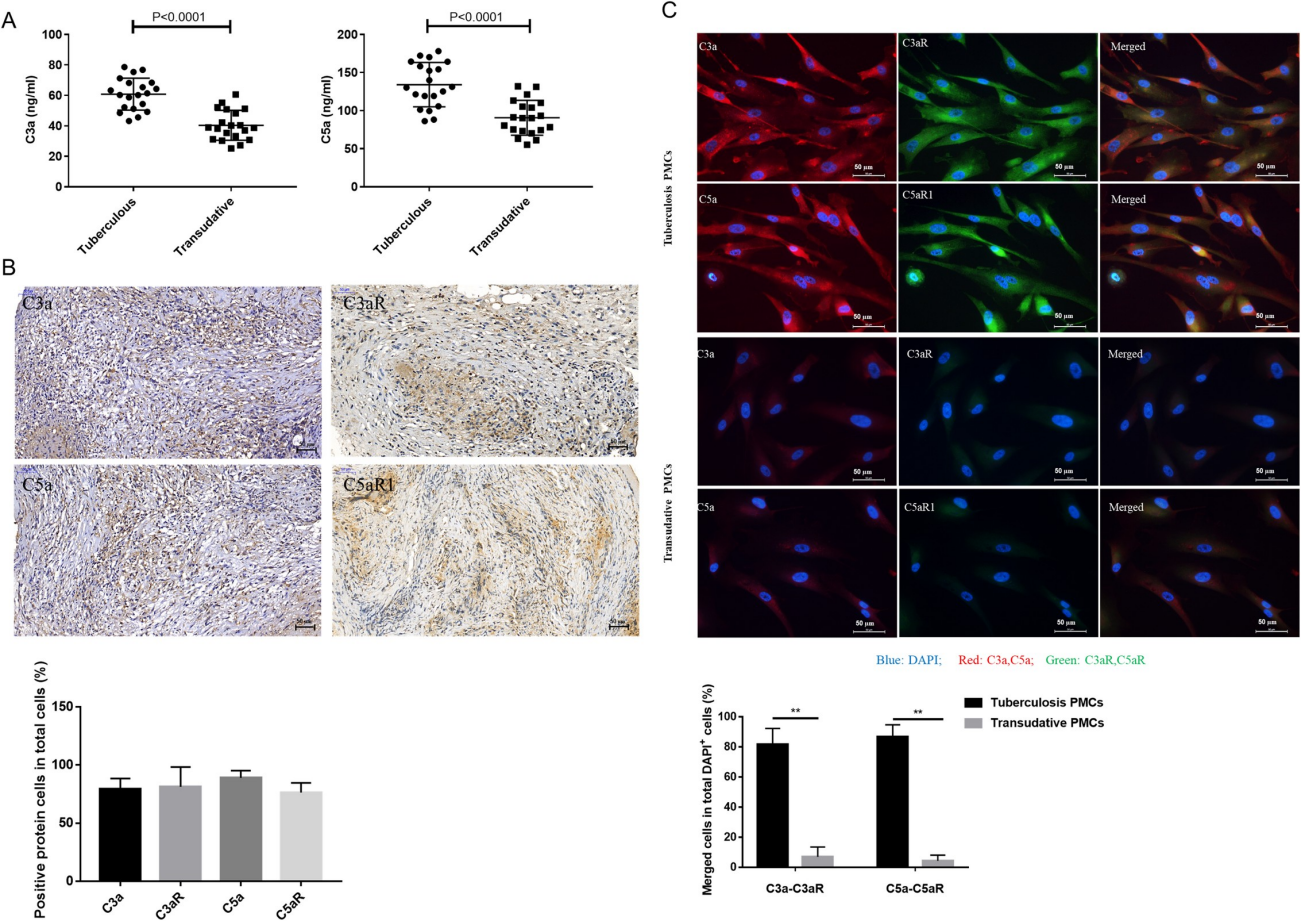
Complement system was activated in the pleural effusion and PMCs of TPE patients. (A) The concentrations of the complement molecules C3a and C5a in pleural effusion were measured by ELISA (n-_tuberculous_ = 20, n-_transudative_ = 20). (B) Representative positive immunohistochemical staining and quantification for anaphylatoxins(C3a and C5a) and their receptors(C3aR and C5aR) in the pleural tissue of TPE patients is shown (original magnification, 200×), (n = 3). (C) Representative intracellular immunofluorescence and quantification on PMCs from TPE or TE with antibodies to C3a(red), C3aR(green), C5a(red), C5aR1(green) and DAPI(blue) (original magnification, 200×), (n = 3), * vs the Transudative PMCs group, ** P<0.01.

Firstly, we reconfirmed that the levels of anaphylatoxins C3a and C5a were higher in TPE than in TE (Fig 1A), which corresponded with our previous research [5]. In addition, immunohistochemistry revealed that anaphylatoxins(C3a and C5a) and their receptors(C3aR and C5aR1) were positive in the pleural tissue of TPE (Fig 1B). Furthermore, we found that the colocalization of anaphylatoxin ligand-receptor pairs was significantly increased in PMCs from patients with TPE compared to those from TE subjects (Fig 1C). Our data indicated that complement signaling was significantly activated in the pleural cavity and PMCs, which led us to explore the roles of the C3a-C3aR and C5a-C5aR1 axes in the pathogenesis of TPE.

### C3a and C5a enhanced cytokine expression and release in PMCs isolated from TPE

**Fig 2:**
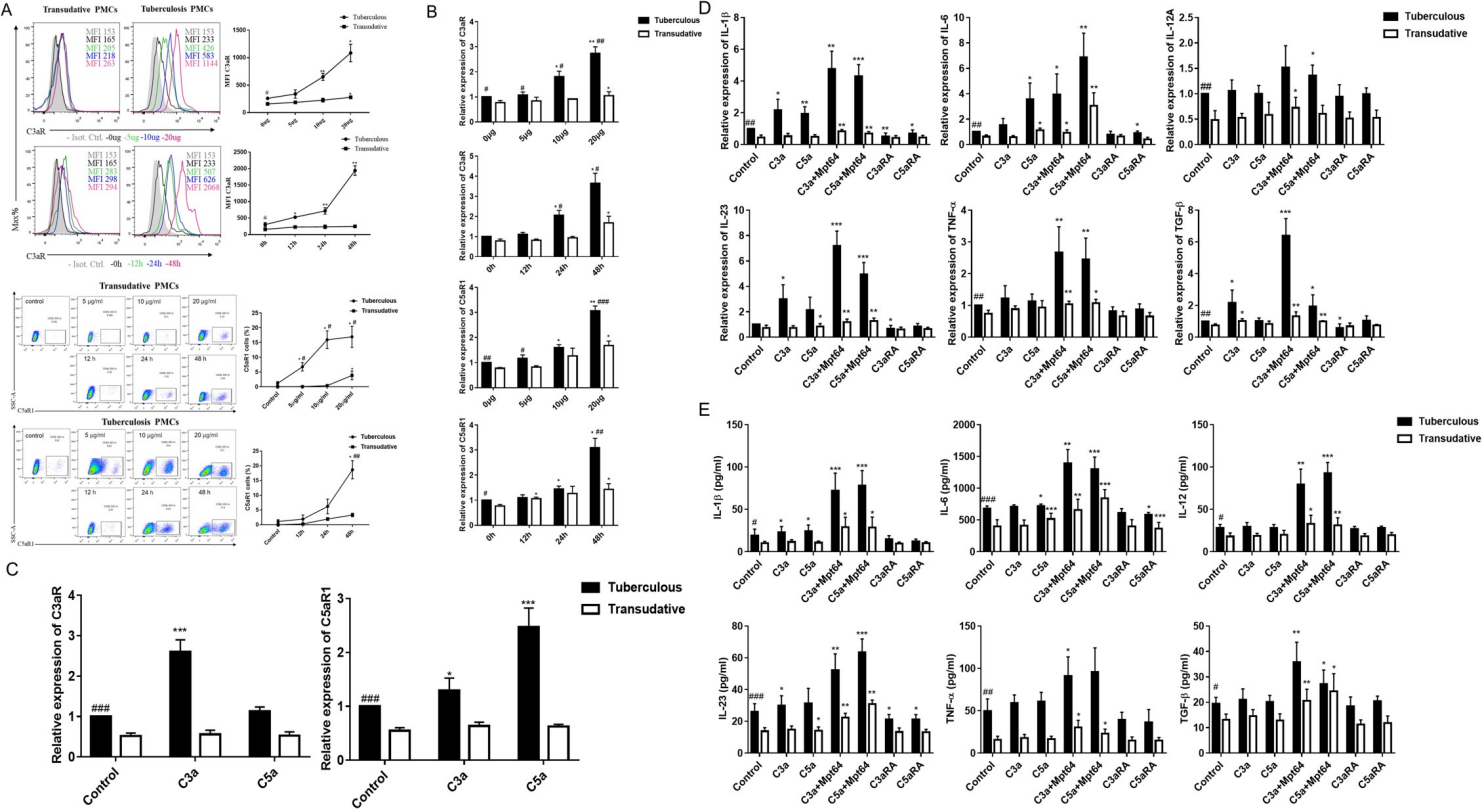
Mpt64 induced the expression of C3aR and C5aR1 in PMCs, and inflammatory cytokine expression and release was upregulated in TPE-PMCs upon anaphylatoxin stimuli. (A-B) PMCs isolated from TPE or TE were incubated with Mpt64 (20 μg/ml) for different times or with different doses of Mpt64 for 24 h. Then, the protein and mRNA expression levels of C3aR and C5aR1 in the PMCs were measured by flow cytometry and RT-qPCR (n = 3). * vs corresponding 0 h group or 0 μg/ml group, * P< 0.05, ** P<0.01; ^#^ vs the Transudative group, ^#^ P< 0.05, ^##^ P<0.01, ^###^ P<0.001,. (C) The expression of C3aR and C5aR1 by PMCs isolated from TPE or TE and treated with C3a (20 nM) or C5a (10 nM) for 24h was measured by RT-qPCR. The expression levels were normalized to those of the housekeeping gene GAPDH (n = 5). * vs corresponding control group, * P< 0.05, *** P<0.001; ^#^ vs the Transudative control group, ^###^ P< 0.01. (D and E) The expression and release of IL-1β, IL-6, IL-12, IL-23, TNF-α and TGF-β by PMCs was measured by RT-qPCR (D) ELISA (E). PMCs were incubated for 24 h in normal medium or medium supplemented with recombinant human C3a (20 nM), recombinant C5a (10 nM), C3a (20 nM)+Mpt64 (20 μg/ml), C5a (10 nM)+Mpt64 (20 μg/ml), C3aR antagonist (50 nM) or C5aR1 antagonist (20 nM) (n = 5). * vs corresponding control group, * P< 0.05, ** P<0.01, ***P<0.001; ^#^ vs the Transudative control group; ^#^ P< 0.05, ^##^ P<0.01, ^###^ P<0.001.

To explore the cause of the increase in the levels of anaphylatoxins and their receptors in PMCs isolated from TPE, we treated PMCs isolated from TPE and TE with the Mtb-derived protein Mpt64. As expected, the expression of both C3aR and C5aR1 protein(Fig 2A) and mRNA (Fig 2B) levels were significantly upregulated in TPE-PMCs upon Mpt64 stimulation in a time- and dose-dependent manner, which corresponded with the C3a and C5a levels increasing in PMCs upon Mpt64 stimulation in our previous study. Moreover, recombinant human C3a and C5a upregulated the expression of the corresponding receptors, C3aR and C5aR1, respectively, in TPE-PMCs (Fig 2C).

Next, we purified PMCs from TPE and TE to identify the cell sources of the elevated cytokine levels in TPE, and the expression of IL-1β, IL-6, IL-12, IL-23, TNF-α and TGF-β in the PMCs isolated from TPE is higher than isolated from TE (Fig 2D and 2E). To evaluate whether anaphylatoxins and Mpt64 have positive influences on cytokine expression and release in PMCs, we used recombinant human anaphylatoxins (C3a and C5a) alone or in combination with Mpt64 to treat the PMCs mentioned above. Real-time RT-PCR (Fig 2D) and ELISA (Fig 2E) results demonstrated that C3a upregulated the level of the cytokines IL-1β, IL-23, TGF-β and C5a upregulated IL-1β and IL-6 from the PMCs isolated from TPE compared with those isolated from TE. However, IL-12 and TNF-α were not affected by C3a or C5a obviously (Fig 2D and 2E). Moreover, anaphylatoxin combined with Mpt64 can up-regulate the expression and release levels of all the above-mentioned cytokines, and the combined group was upregulated at higher levels than the single C3a or C5a. C3aR or C5aR1 antagonists could modestly downregulate increased IL-6, IL-23 at the mRNA and protein levels, respectively. For the PMCs from TE, except for C5a to stimulate IL-6 and IL-23, the above-mentioned anaphylatoxin alone has no obvious effect. However, significant upregulation for all the cytokines in TE when stimulated by Mpt64 in conjunction with anaphylatoxins. (Fig 2D and 2E).

### C3a and C5a enhanced cytokine expression and release in monocytes isolated from TPE

**Fig 3:**
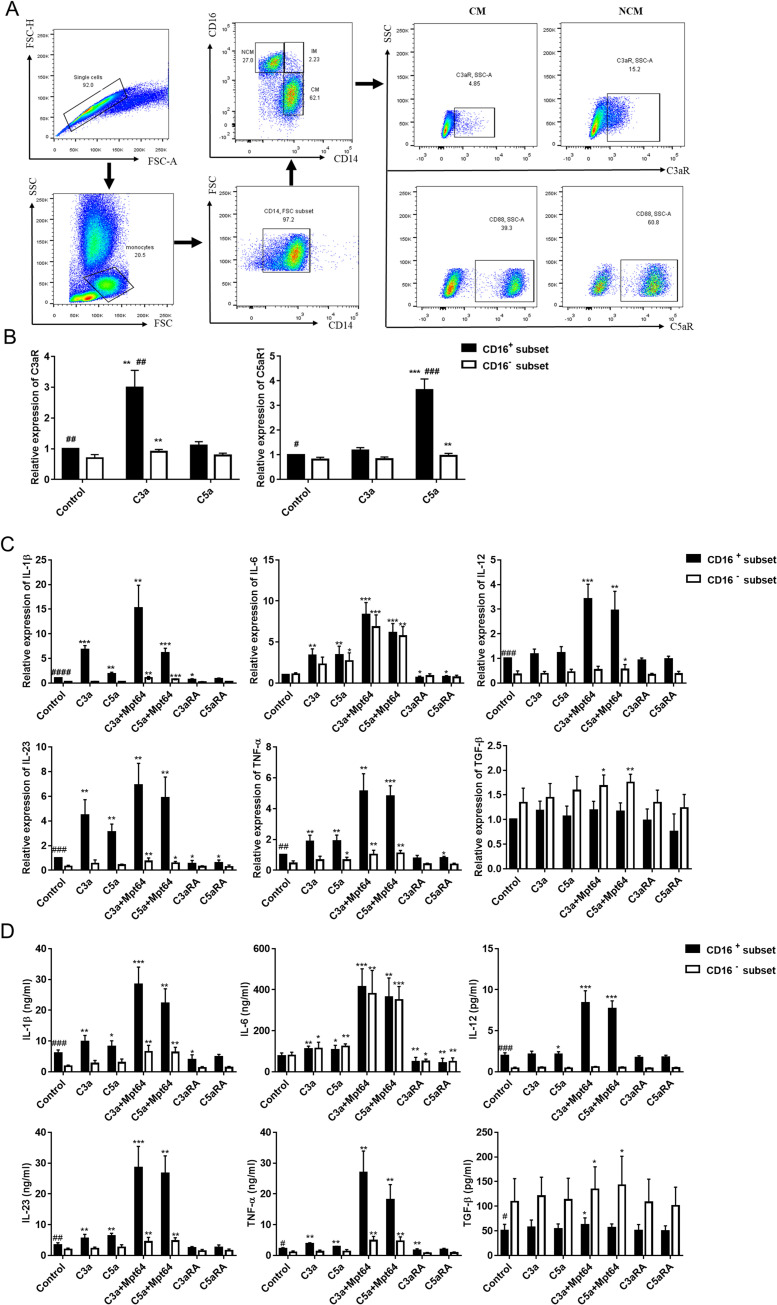
Inflammatory cytokine expression and release was upregulated in monocytes upon anaphylatoxin stimuli. (A) Representative flow charts show the percentages of NCMs, IMs, and CMs from a patient pleural effusion fluids with TPE. NCMs, nonclassical monocytes (CD14+CD16++); IMs, intermediate monocytes (CD14++CD16+); CMs, classical monocytes (CD14++CD16−). (B) The expression of C3aR and C5aR1 by monocytes isolated from patient pleural effusion fluids with TPE and treated with C3a (20 nM) or C5a (10 nM) for 24h was measured by RT-qPCR. The expression levels were normalized to those of the housekeeping gene GAPDH (n = 5). * vs corresponding control group, ** P< 0.01, *** P<0.001; # vs the CD16- subset group, ^#^ P< 0.05, ^##^ P< 0.01, ^###^ P<0.001. (C and D) The expression and release of IL-1β, IL-6, IL-12, IL-23, TNF-α and TGF-β by monocytes from patient pleural effusion fluids with TPE was measured by RT-qPCR (C) or ELISA (D). Monocytes were incubated for 24 h in normal medium or medium supplemented with C3a (20 nM), recombinant C5a (10 nM), C3a (20 nM)+Mpt64 (20 μg/ml), C5a (10 nM)+Mpt64 (20 μg/ml), C3aR antagonist (50 nM) or C5aR1 antagonist (20 nM) (n = 5). * vs the corresponding control group; * P< 0.05, ** P<0.01, *** P<0.001; ^#^ vs the CD16^-^ subset control group; ^#^ P< 0.05, ^##^ P<0.01, ^###^ P<0.001.

In the present study, monocytes isolated from TPE were categorized into two subsets, CD16^+^ monocytes and CD16^-^ monocytes. In humans, CD14^++^CD16^+^ intermediate monocytes (IMs) and CD14^+^CD16^++^ NCMs are collectively considered CD16^+^ subset monocytes (Fig 3A). Similar to PMCs, recombinant human C3a and C5a upregulated the expression of the corresponding receptors, C3aR and C5aR1 in monocytes, especially the CD16^+^ subset (Fig 3B). We found that the expression and release of IL-1β, IL-12, IL-23 and TNF-α was higher in CD16^+^ than in CD16^-^ monocytes isolated from TPE(Fig 3C and 3D). In contrast, the TGF-β level was higher in CD16^-^ than in CD16^+^ monocytes, the IL-6 levels elicited in CD16^+^ monocytes were comparable to those in CD16^-^ monocytes (Fig 3C and 3D). Moreover, C3a and C5a increased the expression and release of the cytokines IL-1β, IL-6, IL-23, TNF-α from the CD16^+^ subset monocytes and of the cytokines IL-6 from the CD16^-^ subset monocytes. (Fig 3C and 3D). C3aR and C5aR1 antagonists could modestly downregulate increased cytokines at the mRNA(Fig 3C) and protein(Fig 3D) levels, respectively. Anaphylatoxin combined with Mpt64 can up-regulate the expression and release levels of almost all the above-mentioned cytokines in two subsets, and the combined group was upregulated at higher levels than the effect of single C3a or C5a. Moreover, the use of C3aR nonpeptide agonist can also achieve similar effects. (S2 Fig)

### Anaphylatoxin-activated monocytes induce Th17 responses

**Fig 4:**
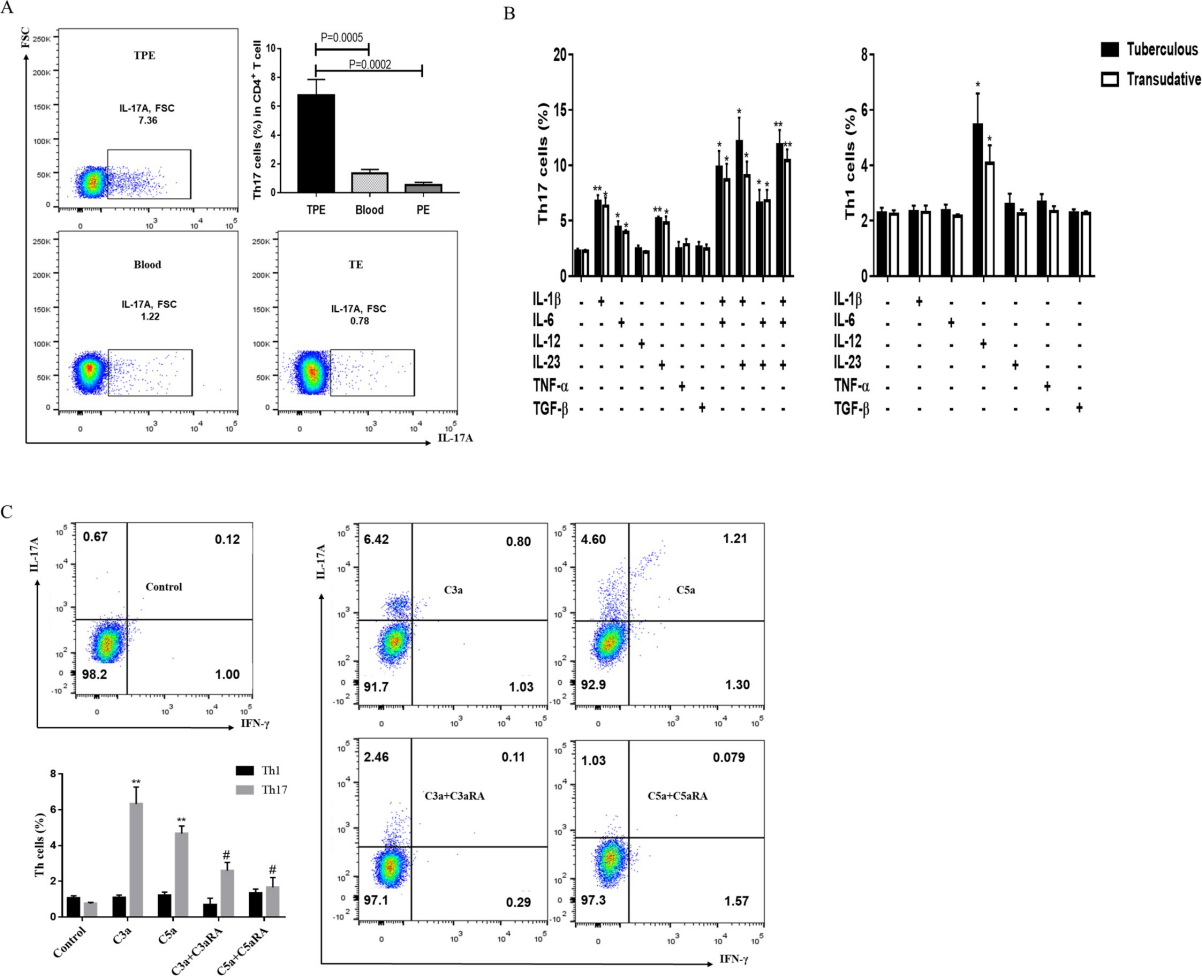
Anaphylatoxin-activated monocytes induce increased Th17 responses. (A) The percentages of Th1 (IFN-γ^+^) and Th17 (IL-17A^+^) cells in TPE, corresponding blood and TE were determined by flow cytometry (n = 5). * vs corresponding blood, ***P<0.001; ^#^ vs transudative group, ^###^ P<0.001. (B) Purified naive CD4^+^ T cells isolated from TPE or TE in the presence of the designated cytokines, either alone or in various combinations and analyzed for IL-17A^+^ and IFN-γ^+^ expression after intracellular staining using the flow cytometry (n = 3). * vs corresponding control group, * P< 0.05, ** P<0.01, ***P<0.001. (C) CD4+ T cells were activated with immobilized Abs to CD3 and CD28 in the presence of supernatants derived from TPE-monocytes that had not been stimulated or stimulated with C3a (20 nM), C5a (10 nM), C3a (20 nM)+C3aRA (50 nM) or C5a(10 nM) +C5aRA (20 nM). Induction of Th17 (IL-17A^+^) and Th1 (IFN-γ^+^) responses was assessed 5 days posttreatment using the flow cytometry. * vs corresponding control group, **P<0.01; ^#^ vs corresponding C3a or C5a group, ^#^ P<0.05.

Considering anaphylatoxin can promote PMC or monocytes to express and secrete cytokines that drive Th17 responses, we investigated the distribution Th17 cells in the peripheral blood of TPE patients and pleural effusion of TPE/TE patients. The results showed that the percentages of Th17(IL-17A^+^) cells in pleural effusion of TPE were significantly higher than those in the blood of TPE patients and pleural effusion of TE patients (Fig 4A), consistent with literature reports [23].

Next, we assessed the above cytokines to differentiate naive CD4^+^ T cells isolated from pleural effusion of TPE and TE. The results showed that IL-1β, IL-6 and IL-23 but not TGF-β were able to promote the expansion of pleural Th17 cells from naïve CD4^+^ T cells, IL-12 but not TNF-α was able to promote the expansion of Th1 cells (Fig 4B). As expected, compared with the corresponding individual cytokines, the combinations of IL-1β plus IL-6, IL-1β plus IL-23, IL-6 plus IL-23, and IL-1β plus IL-6 and IL-23 increased the percentages of Th17 cells to even higher levels (Fig 4B).

Then, we assessed the effects of anaphylatoxin-activated monocytes on CD4^+^ T cell-derived effector cytokine production. In line with expectations, the activation of T cells in the presence of conditioned medium from monocytes pretreated with C3a and C5a demonstrated that the anaphylatoxins induced a significant increase in IL-17A secretion but not IFN-γ(Fig 4C). Moreover, C3aR and C5aR1 antagonists could modestly downregulate the above effect.

### Effects of PMC-monocyte coculture on PMC and monocyte mediator release

**Fig 5:**
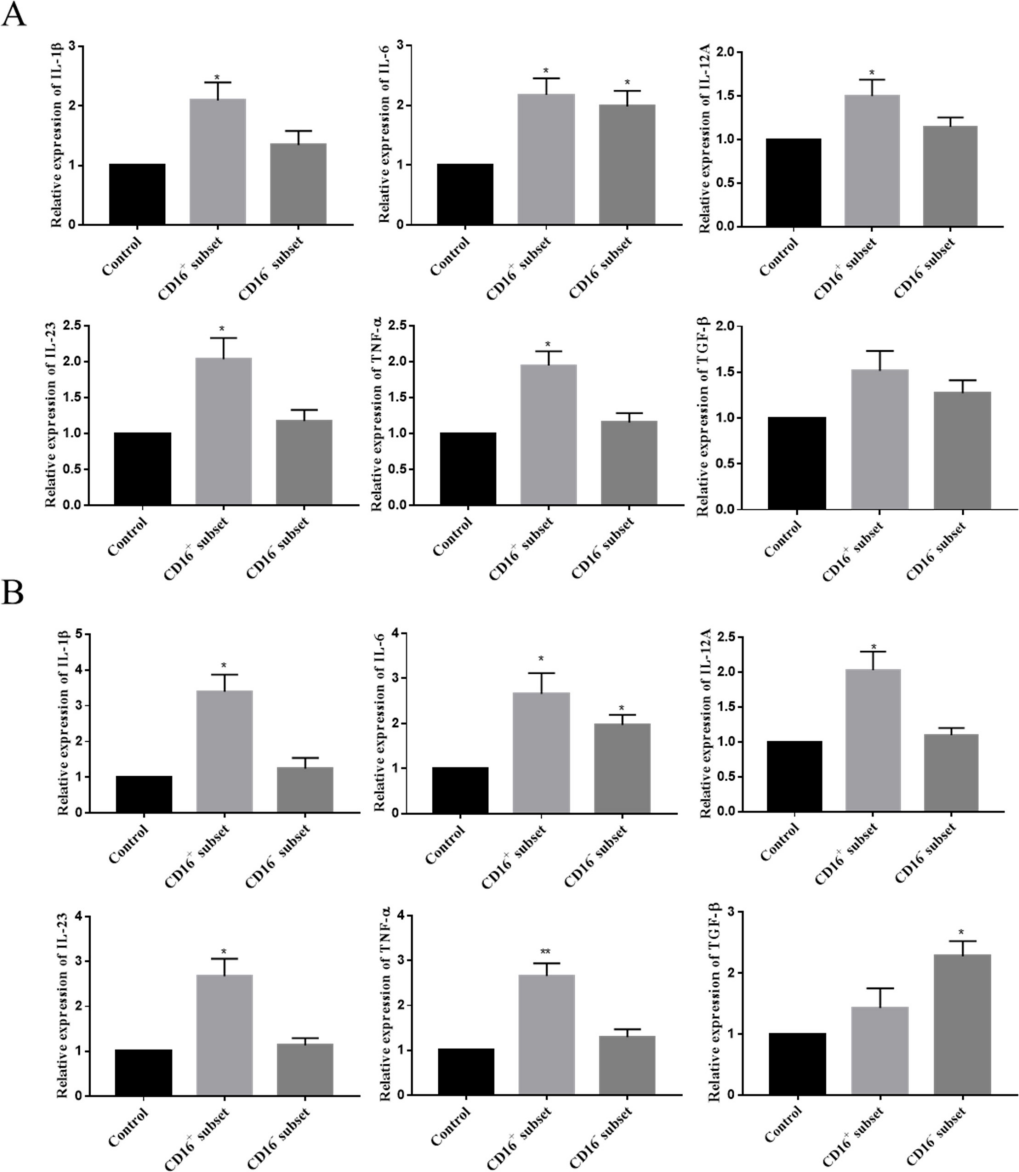
Effect of PMC-monocyte co-culture on monocyte and PMC mediator release. PMCs from TPE were incubated separately with CD16+ monocytes and CD16− monocytes for 24 h. Then, cytokines mRNA expression by PMCs(A) and monocytes(B) were analyzed separately. The expression levels were normalized to those of the housekeeping gene GAPDH (n = 3). * vs the control group, * P< 0.05, ** P<0.01.

We constructed a system for co-culturing PMCs and monocytes and evaluated whether there are interactions between stromal and immune cells in TPE. Firstly, we assessed cocultured-PMCs mediator release. Interestingly, IL-1β, IL-6, IL-12, IL-23 and TNF-α production was upregulated in PMCs cocultured with CD16^+^ monocytes compared to PMCs alone, but CD16^-^ monocytes only upregulated IL-6 expression of PMCs. (Fig 5A). In return, PMCs similarly increased the expression of the cytokines IL-1β, IL-6, IL-12, IL-23 and TNF-α in CD16^+^ monocytes but only increased the expression of IL-6 and TGF-β in CD16^-^ monocytes (Fig 5B).

### Effects of PMC-monocyte coculture on monocyte phenotype

**Fig 6:**
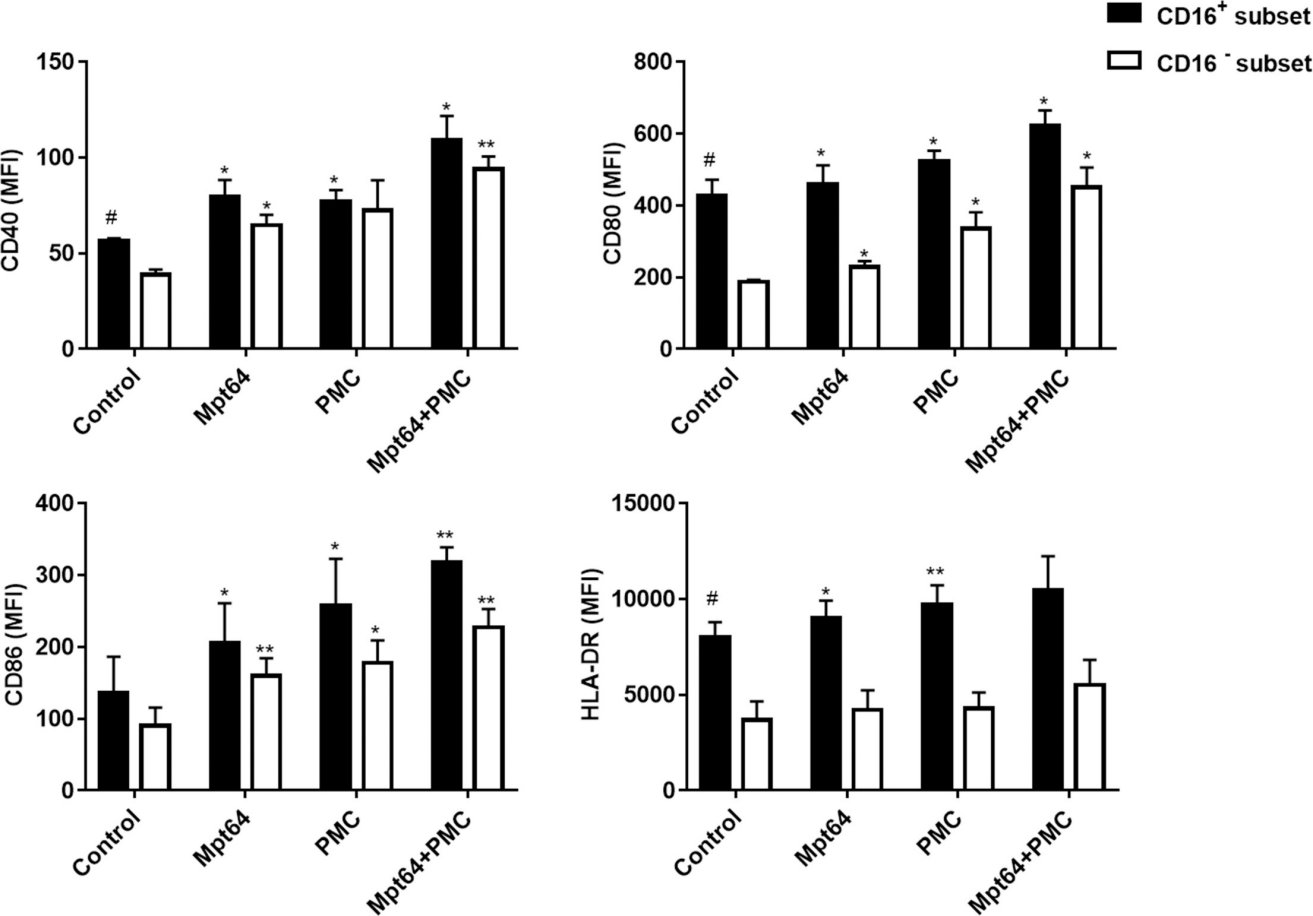
Effect of PMC-monocyte coculture on monocyte phenotype. CD16+ monocytes and CD16− monocytes from the pleural effusion of patients with TPE showed different phenotypes regarding cell-surface markers, including HLA-DR, CD40, CD80, and CD86. MFI, median fluorescence intensity (n = 3). CD16+ monocytes and CD16− monocytes were incubated with PMCs from TPE for 24 h. The surface expression of HLA-DR, CD40, CD80, and CD86 was measured by flow cytometry. Data are presented as the MFI of the whole monocyte population after isotype control subtraction (n = 3). * vs the corresponding control group; * P< 0.05, ** P<0.01; ^#^ vs corresponding CD16- group, ^#^ P<0.05.

We next assessed the phenotypic modulation between the CD16^+^ monocyte subset and CD16^−^ classical monocyte (CM) subset. Firstly, Flow cytometry showed higher expressions of HLA-DR, CD40, CD80, and CD86in CD16^+^ subset than CD16^−^ subset isolated from TPE (Fig 6). We found that the expressions of HLA-DR, CD40, CD80 and CD86 wereup-regulated in any subset of monocytes cocultured with PMCs, which was consistent with the results for direct stimulation of monocytes with Mpt64 group. Moreover, compared with the corresponding individual factors, combinations of Mpt64 plus PMCs increased the expression of costimulatory molecules to even higher levels (Fig 6). Collectively, our results suggest that PMCs and monocytes have some kind of interactions. One of the manifestations of these interactions is that the expression of costimulatory of monocytes can be up-regulated, especially in CD16^+^ monocytes, and this effect is more obvious.

### Anaphylatoxins enhanced the interactions between CD16^+^ monocytes and PMCs

**Fig 7:**
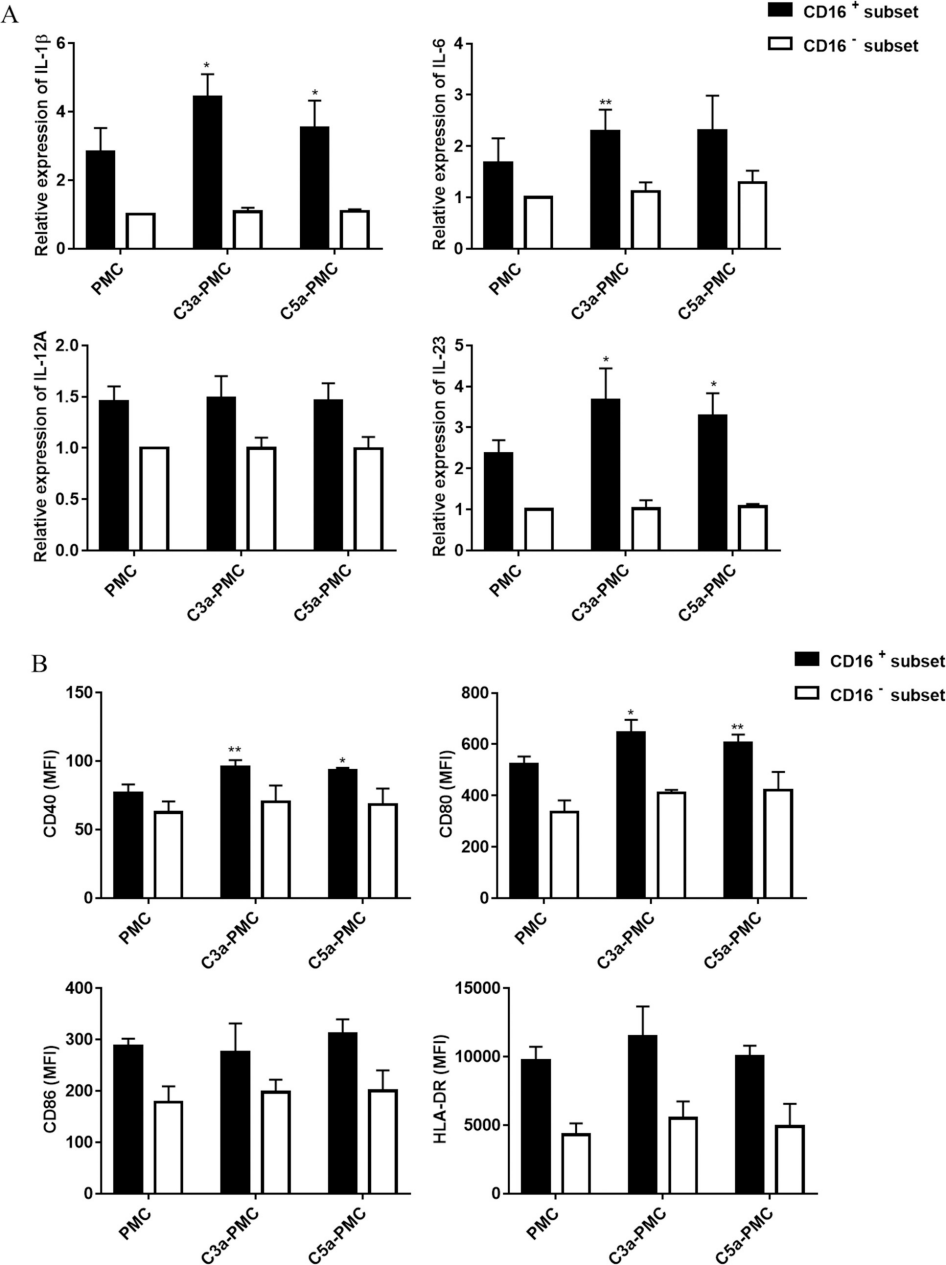
C3a and C5a enhanced the effect of interactions between stromal and immune cells on CD16+ monocytes. PMCs from TPE patients were incubated for 24 h with recombinant human C3a(20 nM), C5a(10 nM). Then, CD16+ monocytes and CD16− monocytes from pleural effusion of TPE were incubated with the pretreated PMCs for 24 h, respectively. (A) The cytokines mRNA expression by monocytes were analyzed separately. The expression levels were normalized to those of the housekeeping gene GAPDH (n = 3). * vs the control group, * P< 0.05, ** P<0.01. (B) The surface expression of HLA-DR, CD40, CD80, and CD86 was measured by flow cytometry. Data are presented as the MFI of the whole monocyte population after isotype control subtraction (n = 3).* vs the corresponding control group; * P< 0.05, ** P<0.01.

Considering that anaphylatoxins can enhance recruitment of nonclassical monocytes via chemokines produced by PMCs in TPE [5], we next evaluated whether anaphylatoxins can enhance the interactions between CD16^+^ monocytes and PMCs. We first used recombinant human C3a and C5a proteins to pre-stimulate PMCs. For CD16^+^ monocytes cytokines, we found that the C3a exposure of PMCs cocultured with monocytes increased the expression of the cytokines IL-1β, IL-6, IL-23 and the C5a exposure of PMCs increased the expression of the cytokines IL-1β, IL-23, but for CD16^-^ cells, the anaphylatoxin exposure of PMC has no effect (Fig 7A). For phenotype, PMCs exposed to C3a and C5a did not modify the CD16^-^ monocyte phenotype but upregulated the expression of CD40 and CD80 in CD16^+^ monocytes (Fig 7B). Collectively, the interactions between monocytes, especially CD16^+^ subset, and PMCs are enhanced by C3a or C5a in TPE.

### Effects of conditioned monocytes on lymphocytes

To assess the effects of interactions between PMCs and monocytes on CD4^+^ T cell responsein TPE, we cocultured naive CD4^+^ T cells with conditioned monocytes previously cultured with anaphylatoxin-pretreated PMCs.

**Fig 8:**
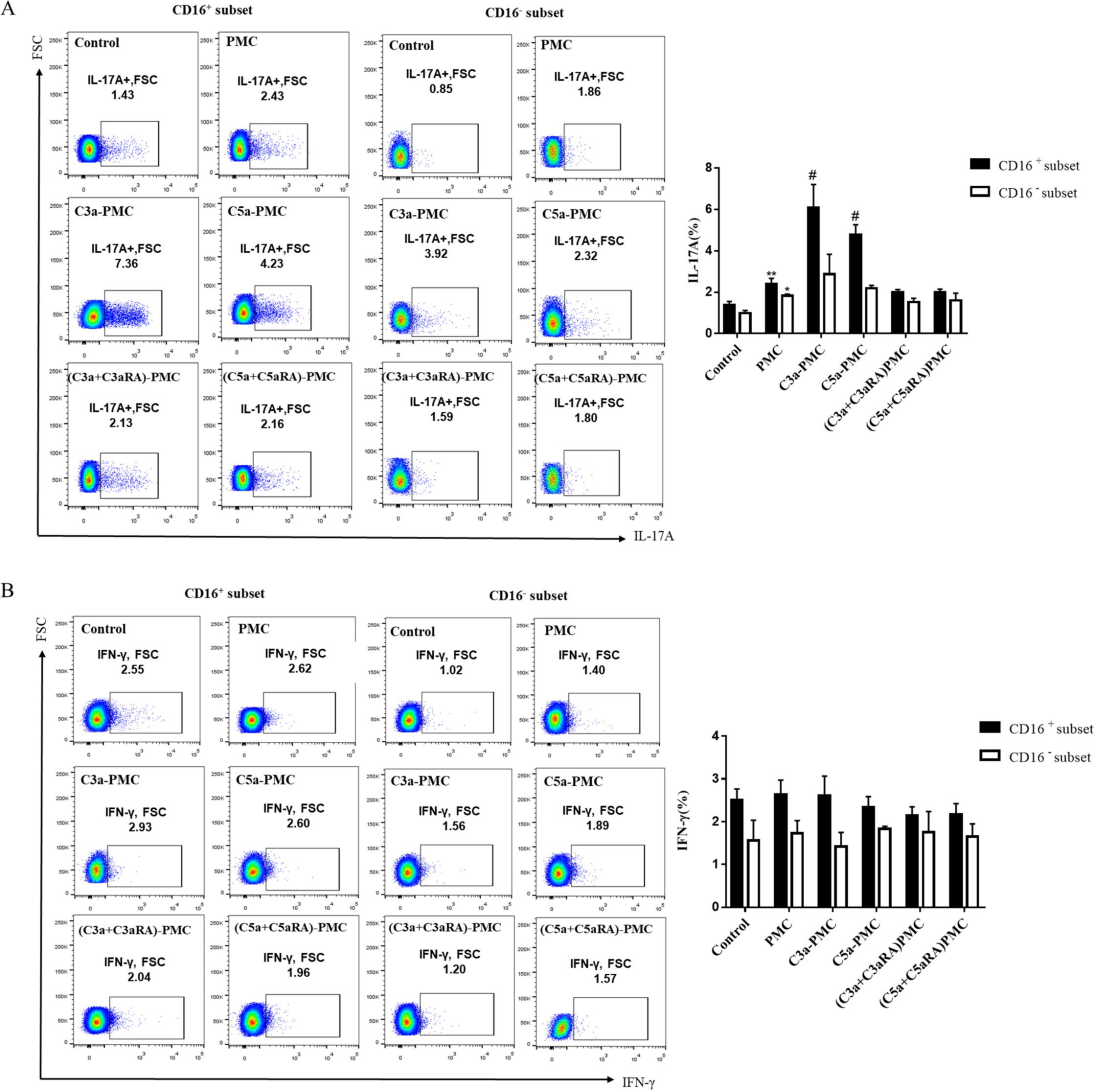
Effect of conditioned monocyte/lymphocyte coculture on Th17 responses. (A and B) PMCs from TPE patients were incubated for 24 h with recombinant human C3a, C5a, C3a+C3aR antagonist or C5a+C5aR1 antagonist. Then, CD16^+^ monocytes and CD16^−^ monocytes from TPE were separately incubated with the primed PMCs for 24 h to form conditioned monocytes, the conditioned monocytes were cocultured with naïve CD4^+^T cells for an additional 5 days, and Th17 (IL-17A), Th1 (IFN-γ) responses were assessed using flow cytometry (n = 3). * vs corresponding control group, ** P<0.01; ^#^ vs corresponding PMC group, ^#^ P< 0.05.

Th17 cells but not Th1 cells were found to occur at an increased frequency in cocultures of CD4^+^ T cells and monocytes conditioned with C3a and C5a treated-PMCs compared to cocultures of control monocytes and CD4^+^ T cells (Fig 8A and 8B).

## Discussion

**Fig 9:**
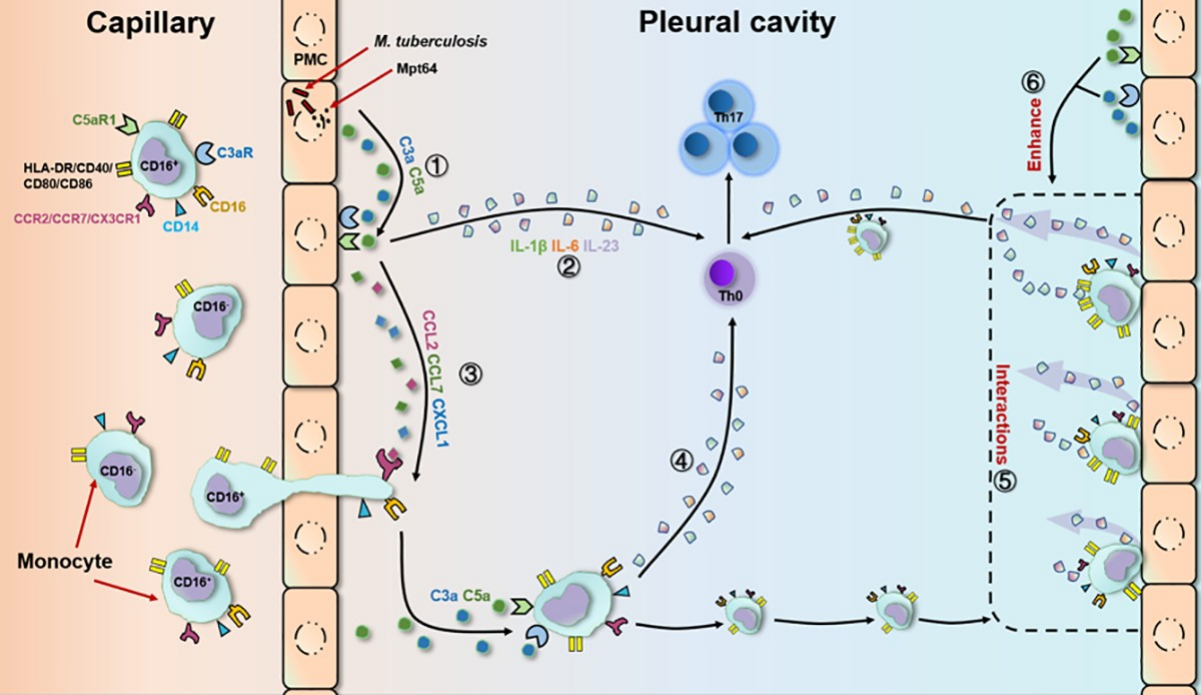
Effect of conditioned monocyte/lymphocyte coculture on Th17 responses. 1. Mpt64 released by Mtb stimulated PMCs to produce anaphylatoxins (C3a and C5a). 2. C3a and C5a promoted the production of cytokines by PMCs, inducing Th17 responses. 3. C3a and C5a induced the production of chemokines by PMCs, resulting in the recruitment of CD16^+^ monocytes to the pleural cavity.[5] 4. C3a and C5a promoted the production of cytokines by CD16^+^ monocytes, inducing Th17 responses. 5. The interactions between monocytes and PMCs enhanced the ability of the PMCs and monocytes to produce cytokines and upregulated the expression of HLA-DR, CD40, CD80 and CD86 on the monocytes, synergistically enhancing the Th17 response. 6. Increased C3a and C5a positively orchestrated the interactions between monocytes and PMCs.

The key findings of this study were as follows: (i) anaphylatoxins (C3a and C5a) induced the production of cytokines by PMCs and CD16^+^ monocytes resulting in Th17 responses but not Th1 responses; (ii) the interactions between monocytes and PMCs enhanced the ability of PMCs to produce IL-1β, IL-6 and IL-23 and upregulated the expression of CD40, CD80, CD86 and HLA-DR by monocytes, synergistically enhancing the Th17 response; and (iii) these effects were orchestrated by increase of anaphylatoxins by PMCs stimulated with Mpt64 (Fig 9). Our results demonstrated that Mtb infection induced the expression of anaphylatoxins and their receptors in PMCs, which subsequently resulted in the amplification of complement activation in TPE.

In the present study, our data has demonstrated that the concentration of C3a and C5a in TPE were higher than TE. In fact, complement activation and function is not confined to the extracellular space but also occurs within cells [24–27], including immune cells(CD4^+^ T cells and monocytes)[28] and stromal cells (human endothelial cells and intestinal epithelial cells) [29–31]. In this study, we found that the expression of anaphylatoxin and its receptors also exist in human pleural mesothelial cells, especially in disease states. For the staining patterns for C3aR and C5aR seem to be distributed perinuclear of PMCs rather than on the cell surface as usual. We think that perhaps anaphylatoxins receptors have been stored in the cell before being transported to the cell surface, or already plays functional roles in intracellular cell sub-compartments[28]. For the specific expression mechanism of anaphylatoxin and its receptor in cells, more research is needed in future work.

There has been a series of evidence indicating that the complement system is involved in mediating T cell activation and expansion[32–35]. However, whether this mechanism exists in TPE is unclear. Th1 cells are essential in the immune response against Mtb. Nevertheless, IFN-γ alone is not sufficient for the complete eradication of the bacteria, suggesting that other cytokines might be required for pathogen removal. The percentage of Th17 cells in pleural effusions of tuberculosis patients was obviously higher than that in peripheral blood mononuclear cells from tuberculosis patients or healthy controls, which is consistent with our results [23, 36]. Th17 cells are important to fight the immune response of extracellular bacteria and fungi. Although Mtb is an intracellular pathogen, Th17 has been reported to be involved in tuberculosis pathology. The proportion of antigen-expanded CD4(+)IFN-γ(+)IL-17(+) lymphocytes, in peripheral blood and pleural fluid from TB patients, was directly correlated with disease severity[37]. In line with this, augmented Th17 response associated with persistent and high antigen load and Mtb drug resistance [38]. Notably, IL-17 is involved in neutrophil recruitment and neutrophils are thought to contribute to the progression of TB[39]. Thus, the involvement of Th17 responses in the pathogenesis of TPE is not surprising. In TPE, the proportions of Th17 cells were correlated positively with Th1 cells suggesting collaboration between Th17 and Th1 cells [36]. Meanwhile, the underlying mechanisms by which Th17 cell numbers are increased in TPE are unclear. In our results, surprisingly, only an increase in the proportion of Th17 cells was observed. We believe that the main reason for this result is the increased proportion of CD16^+^ monocytes and the enrichment of anaphylatoxins in TPE. Meanwhile, the anaphylatoxins in the pleural cavity will further recruit more non-classical monocytes. Compared to CD16^-^ monocytes, CD16^+^ monocytes produce more cytokines that contribute to Th17 polarization, such as IL-1β and IL-23, and the secretion of these cytokines is enhanced again under the stimulation of anaphylatoxins, while IL-12, which is conducive to Th1 polarization, is weak in this situation. Our results indicated that C3a and C5a-activated PMCs and monocytes induced Th17 responses, which corresponds with the literature reporting that C3a and C5a are involved in the modulation of Th17 responses. For example, the specific inducing effects of C3a and C5a on IL-17 cytokine production in both mouse models and human APCs are dependent on the type of APC sensing the C3a and C5a signals and the toll-like receptor (TLR) activated [40, 41]. Furthermore, activation of T cells in the presence of conditioned medium with overexpressed IL-1β, IL-6, and IL-23 from monocytes activated by LPS and a C3aR agonist induced a significant increase in IL-17 secretion, while IFN-γ (Th1) secretion was only marginally increased [20]. However, there is growing evidence that the C5a positively or negatively regulates IL-17. Evidence reveals that C5a limits acute inflammation and antagonizes the IL-17A/IL-23 axis[42], but during endotoxemia maximal production of IL-17F requires complement activation and presence of C5a[43]. Here, we showed that C3a-C3aR and C5a-C5aR1 were both engaged in human PMCs and CD16^+^ monocytes isolated from TPE, which enhanced the release of cytokines such as IL-1β, IL-6, and IL-23, leading to Th17 responses.

This study shows that CD16^+^ monocytes isolated from pleural effusion of TPE patients exhibit a more mature phenotype than CD16^-^ monocytes, such as costimulatory molecules CD40, CD80, CD86 and HLA-DR. Moreover, after co-cultivation with PMCs or Mpt64 treatment, the HLA-DR showed no change on CD16^-^ monocytes, which is consistent with literature reported that HLA-DR is maximally expressed in the middle subset (CD16^+^CD14^++^), while HLA-DR is almost not expressed in CD16^-^ monocytes[44]. On the other hand, the CD16^+^ monocytes exhibit a more mature phenotype than CD16^-^ monocytes and the costimulatory molecules shown up-regulated expression after co-cultivation with pathogenic PMCs or Mpt64 treatment, which is consistent with data showing that in some autoimmune and chronic diseases, CD16^+^ monocytes are enriched and upregulation of anti-inflammatory markers may participate in the induction of inflammatory immune responses[45, 46]. Delphine and colleagues noted that in severe asthma, the interactions between HBECs(human bronchial epithelial cells) and DCs can be established in vitro, promoting DC activation and driving a specific Th response [21]. Similarly, the model of the interactions between PMCs and monocytes in TPE was established in this study. It is worth noting that we chose the Mpt64 antigen during the entire research process because Mpt64 is only secreted from live bacilli, and studies have shown that Mpt64 can be used as a substitute for bacterial viability to diagnose patients with active tuberculosis [47, 48]. We want to simulate the sustained release of proteins from living bacilli by stimulating stromal cells and immune cells to secrete cytokines and up-regulate monocyte costimulatory molecules to affect CD4 ^+^ T cells. We avoided the use of antigens such as ESAT6 that directly stimulate CD4 ^+^ T cells of tuberculosis patients in a MHC class II restricted manner [49].

Subsequently, we used primary human cells obtained from TPE patients, and unlike cell lines in culture, the PMCs and human monocytes retained the patient’s etiology and pathophysiology features in culture. The complex interactions between PMCs and immune cells was modeled by means of a coculture system. Our findings showed that PMCs induced the upregulation of HLA-DR, CD40, CD80 and CD86 expressions in both CD16^-^ and CD16^+^ monocyte subsets, and this upregulation trend was more prominent in the CD16^+^ subset. Additionally, CD16^+^ monocytes were relatively prone to altering their phenotypic pattern, especially by increasing CD40 and CD80 expression, in the presence of anaphylatoxin-prestimulated PMCs. The study demonstrated that anaphylatoxins not only enhanced monocyte activation and their capacity for allostimulation but also promoted their maturation through cell interactions.

In summary, these data highlighted the importance of anaphylatoxins and the innate immune system in eliciting pathogenic T cell responses in TPE and suggested that monocytes, especially the CD16^+^ subset, might be an efficient target for controlling inflammation.

## Supporting information

S1 Fig A. brightfield images of the PMCs. B. Immunofluorescent staining with anti-calretinin antibody of PMCs from the TPE (original magnification, 200×). C-D. purity of CD16+ monocytes, CD16- monocytes and naïve CD4^+^CD45RA^+^ T cells.
(TIF)

S2 Fig Inflammatory cytokine expression and release was upregulated in monocytes upon C3aR nonpeptide agonist. The expression and release of IL-1β, IL-6, IL-12, IL-23, TNF-α and TGF-β by monocytes from patient pleural effusion fluids with TPE was measured by RT-qPCR(A) and ELISA(B). Monocytes were incubated for 24 h in normal medium or medium supplemented with C3aR nonpeptide agonist (C4494) (50 μM), C3aR nonpeptide agonist (C4494) (50 μM)+Mpt64 (20 μg/ml), Mpt64 (20 μg/ml). * vs the corresponding control group; * P< 0.05, ** P<0.01, *** P<0.001; # vs the CD16- subset control group; # P< 0.05, ## P<0.01, ### P<0.001.
(TIF)

S1 Table Characteristics of the tuberculosis and transudative pleural effusion study population. (DOCX)

S2 Table PCR primers. (DOCX)
