# Supplementary figures and images for: Anaphylatoxins orchestrate Th17 response via interactions between CD16+ monocytes and pleural mesothelial cells in tuberculous pleural effusion
Source: PLoS Negl Trop Dis. 2021 Jul 8;15(7):e0009508. doi: 10.1371/journal.pntd.0009508 (PMC8291687; doi:10.1371/journal.pntd.0009508)

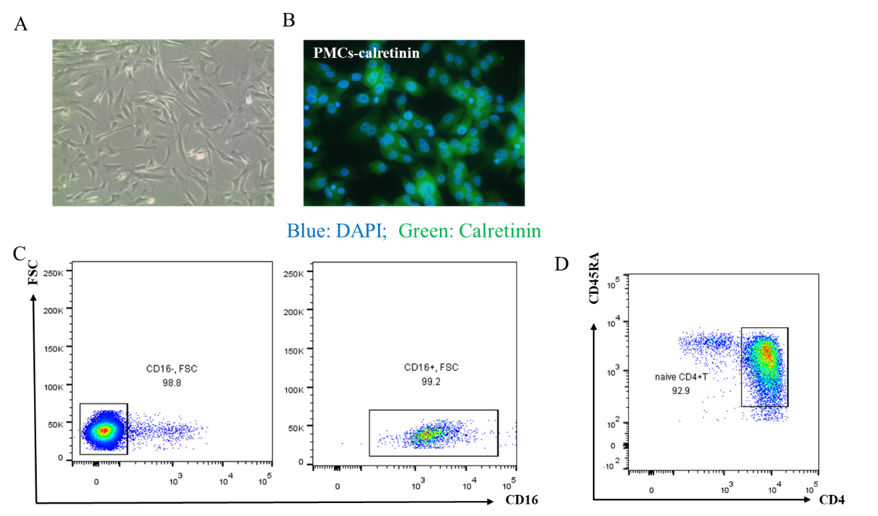

Supplement: S1 Fig — A. brightfield images of the PMCs. B. Immunofluorescent staining with anti-calretinin antibody of PMCs from the TPE (original magnification, 200×). C-D. purity of CD16+ monocytes, CD16- monocytes and naïve CD4+CD45RA+ T cells. (TIF) [file pntd.0009508.s001.tif]

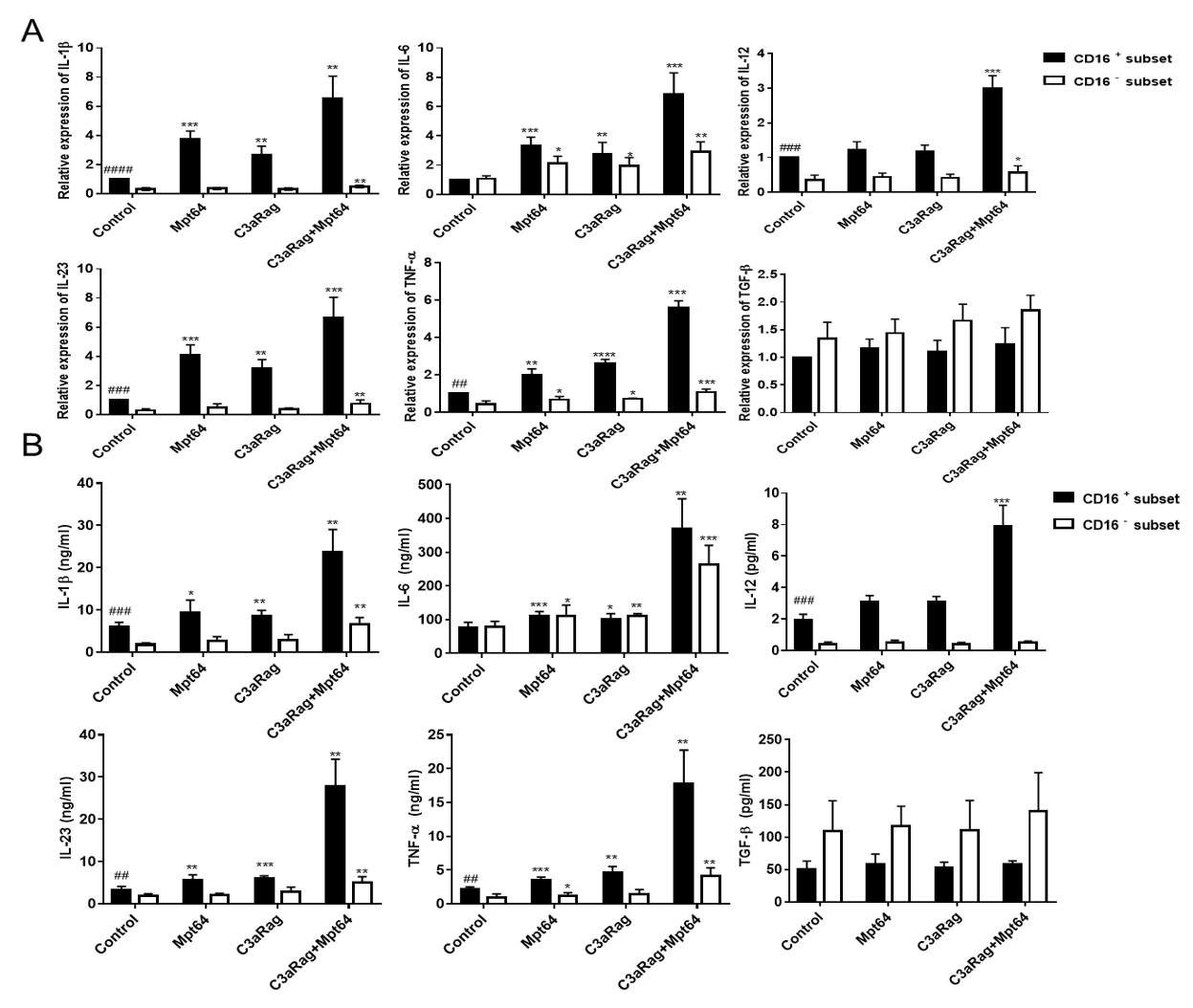

Supplement: S2 Fig — The expression and release of IL-1β, IL-6, IL-12, IL-23, TNF-α and TGF-β by monocytes from patient pleural effusion fluids with TPE was measured by RT-qPCR(A) and ELISA(B). Monocytes were incubated for 24 h in normal medium or medium supplemented with C3aR nonpeptide agonist (C4494) (50 μM), C3aR nonpeptide agonist (C4494) (50 μM)+Mpt64 (20 μg/ml), Mpt64 (20 μg/ml). * vs the corresponding control group; * P< 0.05, ** P<0.01, *** P<0.001; # vs the CD16- subset control group; # P< 0.05, ## P<0.01, ### P<0.001. (TIF) [file pntd.0009508.s002.tif]
