# Supplementary material for: Anaphylatoxins orchestrate Th17 response via interactions between CD16+ monocytes and pleural mesothelial cells in tuberculous pleural effusion
Source: PLoS Negl Trop Dis. 2021 Jul 8;15(7):e0009508. doi: 10.1371/journal.pntd.0009508 (PMC8291687; doi:10.1371/journal.pntd.0009508)
Supplement: S1 Table — (DOCX) [file pntd.0009508.s003.docx]

| Characteristics | Tuberculous | Transudative |
| --- | --- | --- |
| n | 30 | 20 |
| Gender(F/M) | 13/17 | 8/12 |
| Age | 47±7 | 55±8 |
| Sputum culture (+) | 12 | N |
| Blood |  |  |
| Monocytes（*10^9^/L） | 0.71±0.20 | 0.44±0.23 |
| Monocytes%（%） | 9.01±1.66 | 4.21±0.87 |
| Pleural effusion |  |  |
| Monocytes（*10^9^/L） | 1.41±0.37 | 0.44±0.67 |
| Monocytes%（%） | 14.36±2.24 | 5.11±2.01 |
