# Supplementary material for: Anaphylatoxins orchestrate Th17 response via interactions between CD16+ monocytes and pleural mesothelial cells in tuberculous pleural effusion
Source: PLoS Negl Trop Dis. 2021 Jul 8;15(7):e0009508. doi: 10.1371/journal.pntd.0009508 (PMC8291687; doi:10.1371/journal.pntd.0009508)
Supplement: S2 Table — (DOCX) [file pntd.0009508.s004.docx]

|  | Forward | Reverse |
| --- | --- | --- |
| C3aR | CCCTACGGCAGGTTCCTATG | GACAGCGATCCAGGCTAATGG |
| C5aR1 | TCCTTCAATTATACCACCCCTGA | ACGCAGCGTGTTAGAAGTTTTAT |
| IL-1β | ATGATGGCTTATTACAGTGGCAA | GTCGGAGATTCGTAGCTGGA |
| IL-6 | CACTGGTCTTTTGGAGTTTGAG | GGACTTTTGTACTCATCTGCAC |
| IL-12A | CCTTGCACTTCTGAAGAGATTG | GGTCTCTCTGGAATTTAGGCAA |
| IL-23 | TTATGAGAAGCTGCTAGGATCG | GAAGGATTTTGAAGCGGAGAAG |
| TNF-α | AGCTGGTGGTGCCATCAGAGG | TGGTAGGAGACGGCGATGCG |
| TGF-β | CACCGGAGTTGTGCGGCAGT | GGCCGGTAGTGAACCCGTTGAT |
| CD40 | CCTCGCTATGGTTCGTCTGCC | AGCCAGGAAGATCGTCGGGA |
| CD80 | GTGGTCACAATGTTTCTGTTGA | GTTCTTGTACTCGGGCCATATA |
| CD86 | Purchased from Sino Biological, Catalog Number: HP100099 | Purchased from Sino Biological, Catalog Number: HP100099 |
| HLA-DR | AGACAAGTTCACCCCACCAG | TCACCTCCATGTGCCTTACA |
| GAPDH | AGGTCGGTGTGAACGGATTTG | TGTAGACCATGTAGTTGAGGTCA |
